# Activity of the SNARE Protein SNAP29 at the Endoplasmic Reticulum and Golgi Apparatus

**DOI:** 10.3389/fcell.2021.637565

**Published:** 2021-02-18

**Authors:** Elena Morelli, Elisa A. Speranza, Enrica Pellegrino, Galina V. Beznoussenko, Francesca Carminati, Massimiliano Garré, Alexander A. Mironov, Marco Onorati, Thomas Vaccari

**Affiliations:** ^1^Dipartimento di Bioscienze, Università degli Studi di Milano, Milan, Italy; ^2^Dipartimento di Biologia, Unità di Biologia Cellulare e dello Sviluppo, Università di Pisa, Pisa, Italy; ^3^IFOM, The FIRC Institute of Molecular Oncology, Milan, Italy

**Keywords:** SNAP29 gene, Golgi apparatus, endoplasmic reticulum, vesicle fusion, SNARE protein, Syntaxin 5, SEC22B

## Abstract

Snap29 is a conserved regulator of membrane fusion essential to complete autophagy and to support other cellular processes, including cell division. In humans, inactivating *SNAP29* mutations causes CEDNIK syndrome, a rare multi-systemic disorder characterized by congenital neuro-cutaneous alterations. The fibroblasts of CEDNIK patients show alterations of the Golgi apparatus (GA). However, whether and how Snap29 acts at the GA is unclear. Here we investigate SNAP29 function at the GA and endoplasmic reticulum (ER). As part of the elongated structures in proximity to these membrane compartments, a pool of SNAP29 forms a complex with Syntaxin18, or with Syntaxin5, which we find is required to engage SEC22B-loaded vesicles. Consistent with this, in HeLa cells, in neuroepithelial stem cells, and *in vivo*, decreased SNAP29 activity alters GA architecture and reduces ER to GA trafficking. Our data reveal a new regulatory function of Snap29 in promoting secretory trafficking.

## Introduction

Efficient intracellular logistics rely on factors that ensure targeting of the trafficking machinery to membrane compartments. During membrane fusion, long-range delivery is orchestrated by proteins associated to the microtubule cytoskeleton, while docking and tethering factors ensure unambiguous and processive homing at medium range, in hundreds of nanometers away from the destination, as extensively documented during trafficking of vesicles to the endoplasmic reticulum (ER) or within the Golgi apparatus (GA). Finally, a large number of SNARE (Soluble NSF Attachment Receptor) proteins mediate interactions at a short range in association with a multitude of other regulatory factors (for a review, Malsam and Söllner, 2011).

The conserved SNARE protein Snap29 (Soluble NSF Attachment Protein 29) is characterized by the presence of two Q-SNARE domains through which it mediates membrane fusion in association with other Q- and R-SNARE-containing proteins (Steegmaier et al., 1998; Wong et al., 1999; Hohenstein and Roche, 2001). Indeed Snap29 promotes fusion with lysosomes carrying the R-SNARE protein Vamp7 (VAMP8 in humans) (Itakura et al., 2012; Takáts and Juhász, 2013; Morelli et al., 2014). In this process, Snap29 associates first with Syx17 on the surface of the ER or on autophagosomes, likely acting as a Qb-Qc-SNARE, similar to the paralog Snap25. Then, at least in humans, it engages with ATG14 oligomers that act as tethering factors to prime fusion (Itakura et al., 2012; Diao et al., 2015). A number of other less characterized membrane fusion events have been found to involve Snap29, including those occurring during endocytosis and recycling, synaptic transmission, cytokine release, and turnover of secretory granules [for a review, see Mastrodonato et al. (2018)]. Human and *Drosophila* Snap29 also contribute to the formation of the outer part of the kinetochore (KT), which is required to stabilize the plus ends of the microtubule cytoskeleton at the onset of mitosis, ultimately preventing segregation errors and formation of micronuclei (Morelli et al., 2016).

Mutations in *SNAP29* are associated in humans with CEDNIK (cerebral dysgenesis, neuropathy, ichthyosis, and keratoderma), a rare neuro-cutaneous syndrome whose pathogenesis is unclear (Sprecher et al., 2005; Fuchs-Telem et al., 2011). In fibroblasts of CEDNIK patients and in *Snap29* mutant *Drosophila* tissues, the morphology of the GA is altered, suggesting that Snap29 might also play a key role in secretory trafficking (Rapaport et al., 2010; Morelli et al., 2014). Despite a multitude of animal models (Kang et al., 2011; Sato et al., 2011; Schiller et al., 2016; Mastrodonato et al., 2019), the role of Snap29 at the GA and its possible relation to the neuroepithelial traits of CEDNIK have not been elucidated.

Here we explore ER and GA morphology and trafficking upon modulation of Snap9 activity. We show that human SNAP29 forms elongated structures contacting these trafficking compartments and reveals new conserved interactions with the ER and GA Qa-SNAREs Syntaxin 18 (STX18) and Syntaxin 5 (STX5) as well as with the vesicle-associated R-SNARE SEC22B. Interaction with SEC22B, but not with STX18 or STX5, is markedly reduced in a dominant negative SNAP29 mutant that prevents SNARE complex disassembly, suggesting that SNAP29 might initially form a SNARE pre-fusion complex with Qa-SNAREs. Finally, we show that loss of SNAP29 causes defects in GA morphology in human neocortical neuroepithelial stem (NES) cells, an *in vitro* model relevant to neurodevelopmental disorders (Onorati et al., 2016).

## Results

### SNAP29 Supports ER and GA Integrity

**FIGURE 1:**
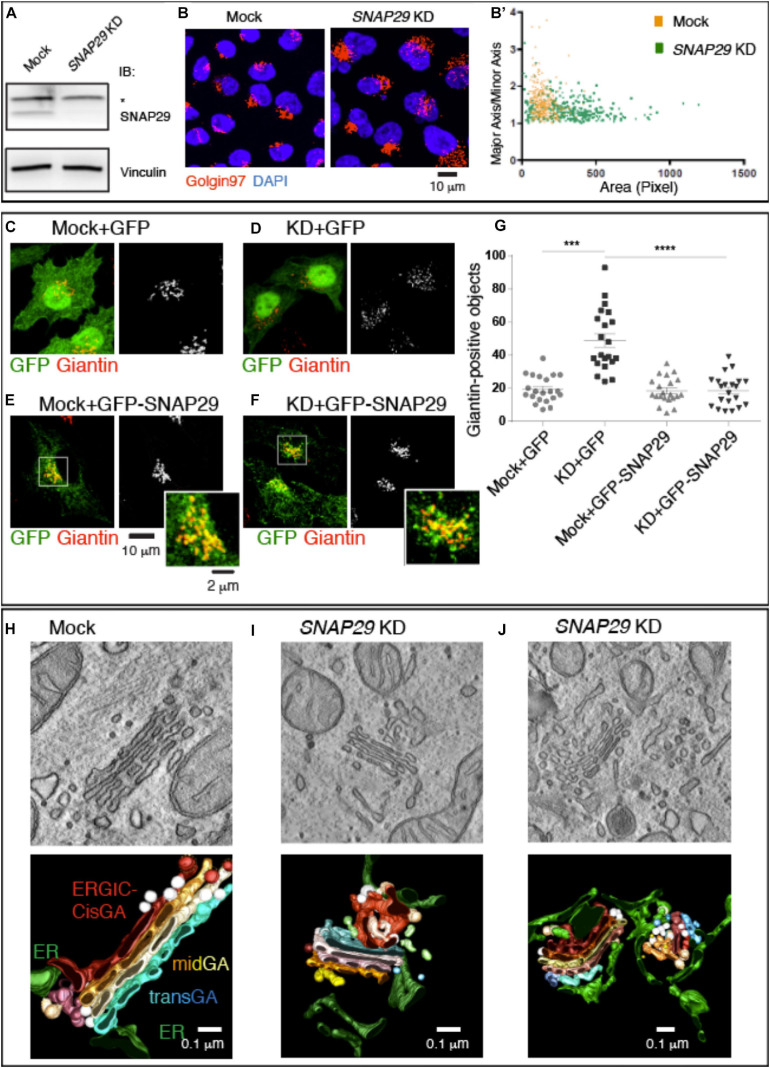
SNAP29 is required to support correct Golgi apparatus (GA) architecture. **(A)** Immunoblotting of total proteins from mock and SNAP29-depleted (KD) HeLa cell protein with the indicated antibodies. The asterisk indicates the presence of a non-specific band recognized by anti-SNAP29. Snap29 is efficiently depleted upon KD. **(B)** Max Projections of mock and SNAP29 KD HeLa cells, stained as indicated. **(B’)** Measurement of the Golgin97-positive area of cells as in panel **(B)**. The area of each measured GA is shown on the *x*-axis, while the ratio of the length of the major axis over the minor axis of the GA is shown on the *y*-axis. Depleted cells display a larger and rounder GA. **(C–F)** Max Projections of mock **(C,E)** and SNAP29 KD HeLa cells **(D,F)** or, in addition, over-expressing the indicated transgene **(E,F)**, stained as indicated. **(G)** Quantification of the number of Giantin-positive objects. The mean with standard error of the mean is shown, and the *p*-value is obtained by one-way ANOVA with Tukey’s multiple-comparisons analysis. GA alterations upon SNAP29 depletion are rescued expression of GFP–SNAP29, which *per se* does not alter GA architecture. **(H–J)** Electron microscopy sections of mock **(H)** and SNAP29-depleted HeLa cells **(I,J)**. 3D tomographic reconstruction of encompassing sections is shown below. In addition to the indicated pseudo-coloring, COPI-coated vesicles are in white, and COPII-coated buds and tubules are in light brown. Clathrin-dependent vesicles are in light blue. SNAP29 depletion leads to GA vesiculation, endoplasmic reticulum (ER)–Golgi intermediate compartment tabulation, and ER enlargement.

Because mutations of SNAP29 result in alteration of the GA architecture in the fibroblasts of CEDNIK patients and in *Drosophila Snap29* mutants (Rapaport et al., 2010; Morelli et al., 2014), we aimed at characterizing in detail the role of SNAP29 at the Golgi apparatus. Compared to mock-treated cells, upon efficient *SNAP29* knock-down (KD) in HeLa cells (Figure 1A), the Golgi apparatus marked by Golgin97 appears round, rather than elongated, and dispersed on a wider area of the cell (Figure 1B, quantified in Figure 1B’). We counted the number of Golgin97-positive objects per cell and found that it increased in *SNAP29* KD relative to mock-treated cells (Figure 1B, quantified in Supplementary Figure S1E), suggesting that the GA is fragmented. A similar phenotype is observed by quantifying the number of objects positive for Giantin, a second GA marker (Figures 1C,D, quantified in Figure 1G). Correct GA morphology is restored upon ectopic expression of a functional RNAi-resistant GFP-tagged form of SNAP29 (GFP–SNAP29; Morelli et al., 2016), which is found enriched at the GA, but not upon expression of GFP alone (Figures 1C–F, quantified in Figure 1G), indicating that SNAP29 is required to support GA architecture. The enrichment of GFP–SNAP29 at the GA recapitulates the earliest Snap29 localization observed (Wong et al., 1999) as well as the *in vivo* localization of a major cellular pool of Snap29 in different tissues of the fruit fly *Drosophila melanogaster* (Morelli et al., 2014; Supplementary Figure S2A), suggesting that SNAP29 might regulate membrane fusion at the GA.

To assess whether the role of SNAP29 in supporting GA architecture is conserved, we also expressed in HeLa cells a CFP-tagged form of *Drosophila* Snap29 (CFP-Snap29), capable of rescuing the loss of *Drosophila* Snap29 (Morelli et al., 2014), which also displays localization to GA *in vivo* (Supplementary Figure S2B). Similar to GFP–SNAP29, the expression of CFP-Snap29 in *SNAP29* KD HeLa cells rescues GA morphology (Supplementary Figures S1A–D, quantified in Supplementary Figure S1E). Importantly, the expression of GFP–SNAP29 or CFP-Snap29 for a short time (see “MATERIALS AND METHODS”), *per se*, does not alter GA morphology (Figure 1E, quantified in Figure 1G and Supplementary Figures S1A,C, quantified in Supplementary Figure S1E). As in *SNAP29* KD HeLa cells, GA disruption is also observed in a fibroblast cell line depleted with the same siRNA used for HeLa experiments (Supplementary Figures S2C,D).

To better characterize the morphology of the GA in the absence of SNAP29, we performed electron microscopy (EM) and 3D tomography reconstruction (Figures 1H–J). Consistent with immunofluorescence data, compared to mock-treated control (Figure 1H), *SNAP29* KD cells display multiple alterations. The GA cisternae are deformed, enlarged, and often replaced by anastomosed tubular structures surrounded by COPI- and COPII-coated vesicles, and the ER–Golgi intermediate compartment (ERGIC) as well as the ER surrounding the GA area are enlarged (Figures 1I,J; quantification of the cisternal width is shown in Supplementary Figure S1F). In extreme cases, the cisternae are in part replaced by vesicles of different sizes surrounded by an aberrantly expanded and reticulated ER (Figure 1J). These data indicate that SNAP29 is required to maintain the integrity of GA, ERGIC, and ER and suggest that SNAP29 might regulate vesicle trafficking and membrane fusion between these compartments.

Overall, our evidence indicates that the localization of SNAP29 to the GA and its role supporting GA architecture are conserved and not cell-type specific.

### A Pool of GFP–SNAP29 Localizes in Elongated Structures Close to Golgi Cisternae and ERGIC Compartments

**FIGURE 2:**
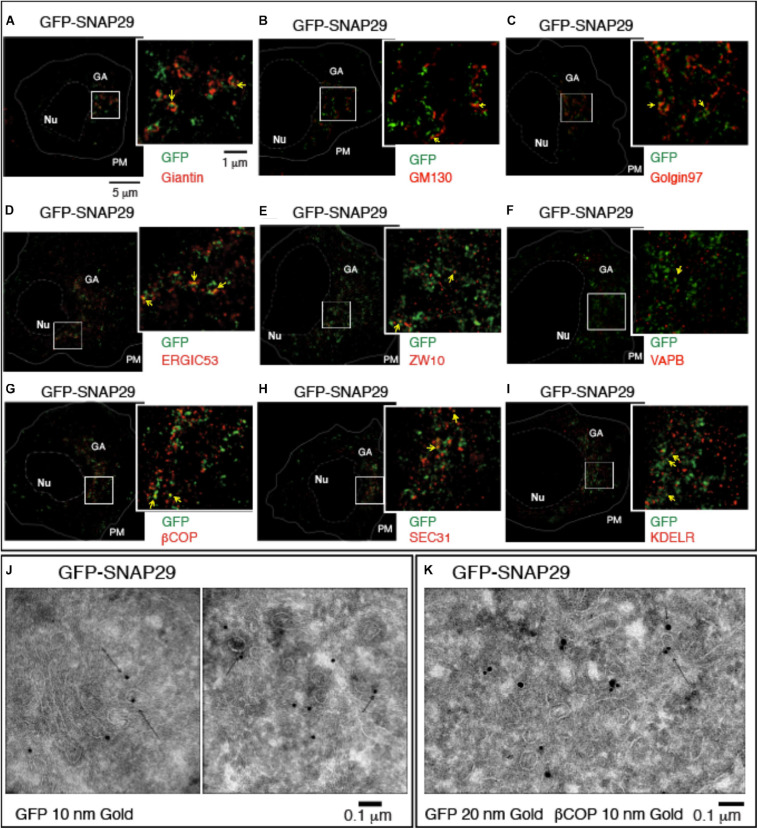
GFP–SNAP29 partially colocalizes with endoplasmic reticulum (ER) and Golgi apparatus (GA) markers. **(A–I)** Single sections of HeLa cells over-expressing GFP–SNAP29 for 6 h stained as indicated and acquired by stimulated emission depletion microscopy. The dashed and the continuous lines delimit the nucleus and the plasma membrane, respectively. The boxed GA area is magnified in the insets. Yellow arrows point to an example of co-localization between GFP–SNAP29 and ER or GA markers. **(J,K)** Cryo-immuno-EM sections of HeLa cells stably transfected to express GFP–SNAP29, stained, and revealed as indicated. Some GFP–SNAP29 localize to the ERGIC area and colocalizes with a COPI marker.

To understand how SNAP29 might act to maintain the structure of the GA, ERGIC, and ER, we next studied the localization of GFP–SNAP29 in proximity of the GA by stimulated emission depletion (STED) microscopy in HeLa cells. Intriguingly, in single sections of super-resolution images, we find that GFP–SNAP29 forms of elongated and often branched structures, 100 to 500 nm in length, in proximity of the GA cisternae marked by Giantin (Figure 2A). The extremities of such GFP–SNAP29 structures partially overlap with Giantin, with GM130, a marker of the cis-Golgi compartment, or with Golgin97 (Figures 2A–C). The partial co-localization of the extremities of GFP–SNAP29 structures is also observed with ERGIC53, a component of the ERGIC compartment (Figure 2D), or the ER component ZW10 (Hirose et al., 2004; Figure 2E). Some limited proximity is observed with the vesicle-associated membrane protein (VAMP)-associated protein B (VAPB), which anchors the ER membranes to microtubules for stability (Amarilio et al., 2005; Figure 2F). These data suggest that GFP–SNAP29 structures contact the area comprised between the ER and the GA.

To investigate the relationship between SNAP29 and ER–GA trafficking, we next assessed the colocalization of GFP–SNAP29 trafficking markers. To this end, we stained cells for βCOP, a component of COPI membrane coats, and for SEC31, a marker of COPII coats, which initiate retrograde and anterograde transport between ER and GA, respectively. Interestingly, we observed that portions of the GFP–SNAP29 structures often co-localize with both βCOP and SEC31 (Figures 2G,H) and with the ER recycling receptor KDELR (Figure 2I). In agreement with super-resolution data by Cryo-EM, we observed GFP localization at the ERGIC compartment, in proximity of the Golgi cisternae (Figure 2J), and colocalization with membranes marked with βCOP (Figure 2K), indicating that SNAP29 might participate in membrane fusion at the ER and GA.

### SNAP29 Depletion Delays Cargo Trafficking Between the ER and GA

**FIGURE 3:**
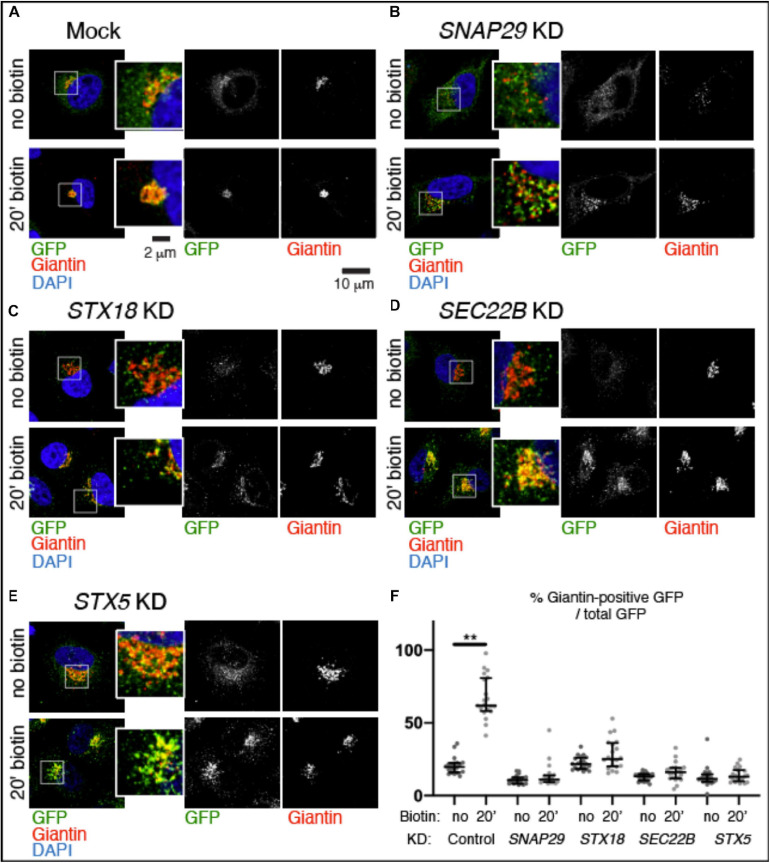
SNAP29 contributes to ManII–SBP–GFP trafficking to the Golgi apparatus (GA). **(A–E)** Single confocal sections of HeLa cells stably expressing ManII–SBP–GFP, treated and stained as indicated. The EGFP pattern has been imaged before addition of biotin (no biotin) and 20 min after addition of biotin (20 min biotin). The insets show close-ups of the GA and surrounding areas. **(F)** Quantification of the ratio of the Giantin-positive EGFP signal over total, relative to the experiment in panel **(A–E)**. SNAP29, as well as the endoplasmic reticulum and GA SNAREs STX18, STX5, and SEC22B, appears to support ManII–SBP–GFP trafficking to the GA. The median with interquartile range is shown, and the *p*-value is obtained by Dunn’s multiple-comparisons test.

To test whether trafficking is affected by depletion of SNAP29, we followed transport from the ER to the GA of a GFP-tagged form of the reporter Mannosidase II fused with streptavidin binding protein (SBP) (ManII–SBP–GFP) in HeLa cells. As part of the RUSH system, ManII–SBP–GFP is retained in the ER until biotin is added to allow trafficking of the reporter to the GA (Boncompain et al., 2012). Consistent with this, in mock-treated cells, ManII–SBP–GFP poorly localizes to Golgin97- or Giantin-positive perinuclear Golgi area, and most of the EGFP signal is dispersed (Supplementary Figure S3A, no biotin; Figure 3A, no biotin, quantified in Figure 3F). In contrast, at 20 min after biotin addition, most ManII–SBP–GFP colocalized with Golgin97 or Giantin, indicating that a significant portion of the reporter has reached the GA (Supplementary Figure S3A, no biotin; Figure 3A, 20 min biotin, quantified in Figure 3F). While in HeLa cells efficiently depleted of SNAP29 the number of Golgin97- or Giantin-positive objects is increased due to GA fragmentation, in the absence of biotin, ManII–SBP–GFP poorly colocalizes with Golgin97 or Giantin, similar to what we observed in mock-treated controls (Supplementary Figure S3B, no biotin; Figure 3B, no biotin, quantified in Figure 3F). However, in contrast to mock-treated controls, in *SNAP29* KD cells, ManII–SBP–GFP colocalization with Golgin97 or Giantin is not significantly increased 20 min after addition of biotin, indicating that a large portion is unable to reach the GA (Supplementary Figure S3B, 20 min biotin; Figure 3B, 20 min biotin, quantified in Figure 3F). Despite this, in both mock-treated and *SNAP29* KD cells, we observed full colocalization of ManII–SBP–GFP with Golgin97 at 1 h after addition of biotin, suggesting that trafficking from ER to GA is delayed in the presence of reduced amounts of SNAP29 (Supplementary Figures S3A,B, 60’ biotin). A similar delay is visible upon downregulation of the SNARE Syntaxin18 (STX18; Figure 3C, 20 min biotin, quantified in Figure 3F), which regulates fusion of the incoming vesicles at the ER and cis-GA (Hatsuzawa et al., 2000) as well as by depleting SEC22B (Figure 3D, 20 min biotin, quantified in Figure 3F), which is carried by retrograde and anterograde trafficking vesicles (Aoki et al., 2008) or STX5 (Figure 3E, 20 min biotin, quantified in Figure 3F), which is required for fusion of anterograde cargoes on the surface of GA cisternae (Hay et al., 1997; Xu et al., 2000). The levels of depletion for each SNARE protein are shown in Supplementary Figure S3C, while Supplementary Figure S3D reports the observed disruption of GA architecture upon depletion of each SNARE. Overall, these data indicate that SNAP29 activity might contribute to vesicle transport between the ER and the GA.

### SNAP29 Interacts With SNAREs at GA and ER Membranes

**FIGURE 4:**
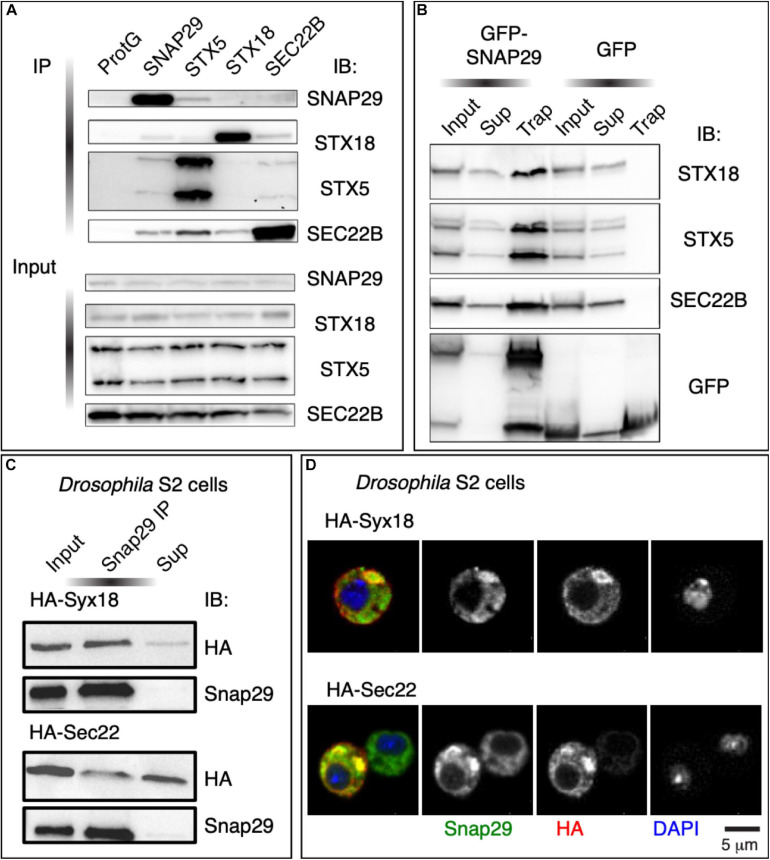
SNAP29 interacts with the endoplasmic reticulum (ER) and the Golgi apparatus (GA) SNAREs. **(A)** Immunoblotting of proteins immunoprecipitated from HeLa protein extracts with the indicated antibodies and related inputs. Endogenous SNAP29 interacts with the ER and GA SNAREs STX18, STX5, and SEC22B. **(B)** Immunoblotting of proteins immunoprecipitated using GFP Trap from protein extracts of HeLa cells expressing GFP–SNAP29 or GFP as a control and related inputs and supernatants. GFP–SNAP29 interacts with the ER and GA SNAREs. **(C)** Immunoblotting of protein extracts from *Drosophila* S2 cells over-expressing the indicated transgenes immunoprecipitated with the indicated antibody and related controls. Endogenous *Drosophila* Snap29 interacts with HA-Syx18 and HA-Sec22. **(D)** *Drosophila* S2 cells over-expressing the indicated transgenes and stained as indicated. Endogenous *Drosophila* Snap29 colocalizes with HA-Syx18 and HA-Sec22.

To identify the steps at which SNAP29 might act in ER–GA trafficking, we immunoprecipitated endogenous SNAP29 from HeLa cell total protein extract and tested whether STX5, STX18, and SEC22B are found as co-precipitants. Interestingly, we found STX5, STX18, and SEC22B in complex with SNAP29 (Figure 4A), and we confirmed the interactions by precipitating GFP–SNAP29 from expressing cells using the GFP–Trap system (Figure 4B). As expected, we did not find an interaction between STX5 and STX18, and we confirmed known interactions between STX5 and SEC22B and between STX18 and SEC22B (Figures 4A,B). Consistent with protein–protein interaction results, by super-resolution microscopy, we observed that GFP–SNAP29 structures partially overlap with STX5, STX18, and SEC22B (Supplementary Figure S4A). Interaction and colocalization of endogenous Snap29 with HA-tagged Syx18 or Sec22 can also be observed in *Drosophila* S2 cells (Figures 4C,D). Overall, these data indicate that SNAP29 possess a conserved ability to associate with known GA and ER SNAREs.

### SNAP29 Regulates Membrane Fusion by Forming a Precomplex With Qa-SNAREs

**FIGURE 5:**
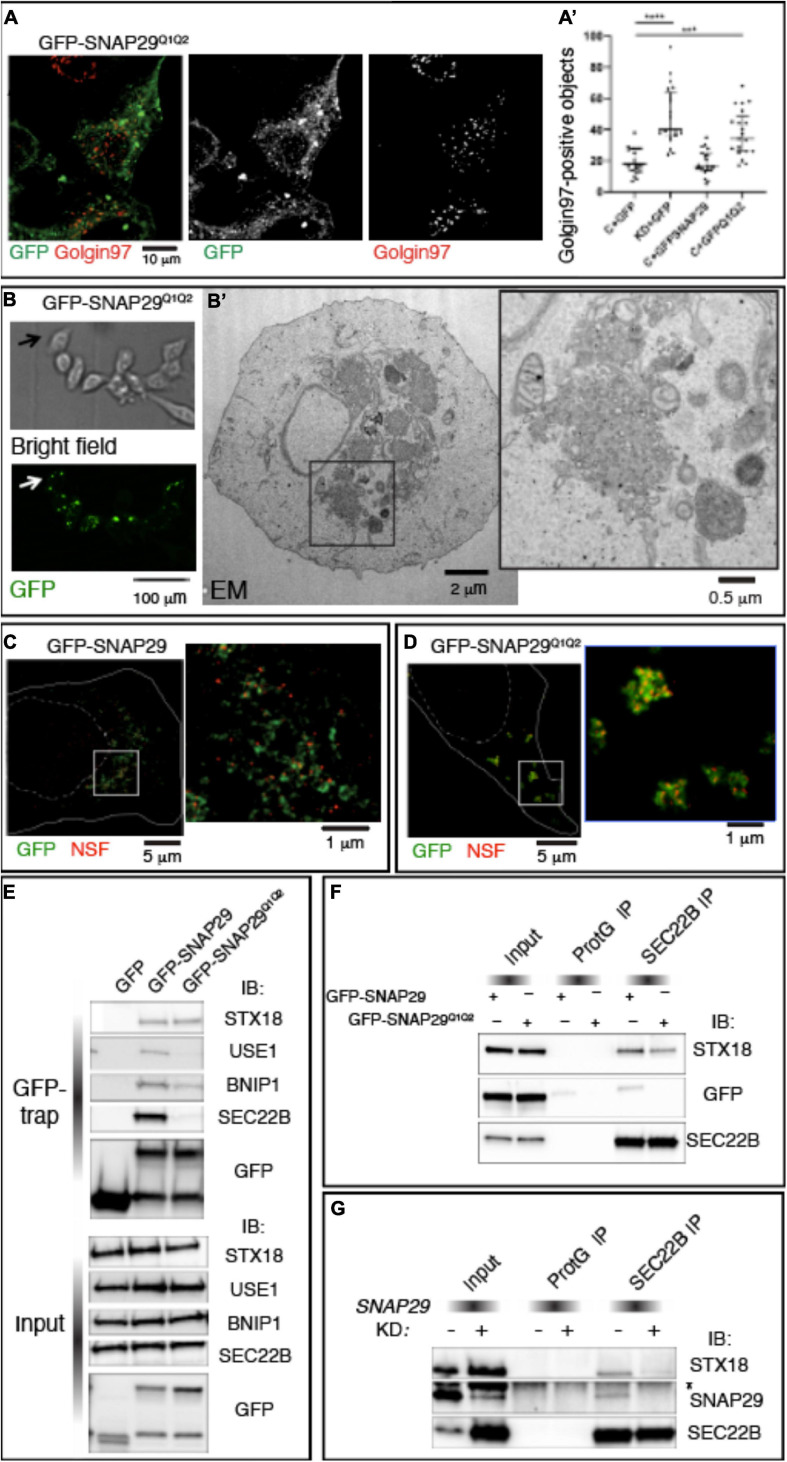
SNAP29 interacts primarily with STX18 and is required to stabilize interactions with SEC22B. **(A)** HeLa cells expressing GFP–SNAP29^Q1Q2^. Cells stained with anti-Golgin97 to mark the Golgi apparatus (GA) show that GFP–SNAP29^Q1Q2^ forms enlarged bodies at the cell periphery and that the GA is fragmented. **(A’)** Quantification of Golgin97-positive GA object upon expression of the indicated transgenes reveals that GFP–SNAP29^Q1Q2^ induces GA fragmentation, thereby acting as a dominant negative SNAP29 form. The mean with standard error of the mean is shown, and the *p*-value is obtained by one-way ANOVA with Tukey’s multiple-comparisons analysis. **(B)** Representative images of a CLEM analysis of a HeLa cell expressing GFP–SNAP29^Q1Q2^. Single sections of HeLa cells expressing GFP–SNAP29^Q1Q2^ collected at phase contrast (bright field) and by confocal microscopy (GFP) to visualize the cell morphology and GFP–positive bodies. **(B’)** Electron microscopy image of the cell indicated by the arrow in panel **(B)**. The GFP–SNAP29^Q1Q2^ bodies are composed of vesicular material and fragmented GA cisternae as highlighted in a close-up of the cytoplasmic portion boxed in panel **(B’)**. **(C,D)** Single sections of HeLa cells over-expressing the indicated SNAP29 forms stained as indicated and acquired by stimulated emission depletion microscopy. The dashed and the continuous lines delimit the nucleus and the plasma membrane, respectively. High magnifications of the boxed areas are shown in the insets. The GFP–SNAP29^Q1Q2^ bodies are highly decorated with N-ethylmaleimide-sensitive fusion. **(E)** Immunoblotting (IB) with the indicated antibodies of proteins immunoprecipitated using GFP Trap from protein extracts of HeLa cells expressing the indicated transgenes and related control. Interactions with Qb, Qc, and R-SNAREs, but not with Qa-SNARE STX18, are weakened by the expression of GFP–SNAP29^Q1Q2^. **(F,G)** IB with the indicated antibodies of protein extracts from HeLa cells over-expressing the indicated transgenes **(F)** or treated as indicated **(G)** and immunoprecipitated with anti-SEC22B and related controls. The asterisk indicates a non-specific band recognized by anti-SNAP29. GFP–SNAP29^Q1Q2^ is not included in SEC22B immunoprecipitations, and SNAP29 depletion impairs the interaction of SEC22B with STX18.

To uncover the mechanism by which SNAP29 regulates membrane fusion, we studied HeLa cells expressing GFP–SNAP29^Q1Q2^, a SNAP29 in which we mutated to Ala (A) each of the two central Gln (Q) of the SNARE domains (Morelli et al., 2016). GFP–SNAP29^Q1Q2^ localizes and acts radically different from GFP–SNAP29. In fact, GFP–SNAP29^Q1Q2^ is not localized close to the GA area but rather accumulates in large bodies at the cell periphery, and it causes GA fragmentation *per se* (Figures 5A,A’).

To determine the morphology of the large bodies induced by the expression of GFP–SNAP29^Q1Q2^, we performed correlative light electron microscopy (CLEM; Figure 5B). We observed that GFP–SNAP29^Q1Q2^ bodies appear constituted by clusters of vesicles of different sizes (Figure 5B’). Such organization replaces entirely ER and GA structures and is similar to extreme cases of *SNAP29* depletion. Consistent with a possible vesiculation of the ER, ERGIC, and GA membranes, GFP–SNAP29^Q1Q2^ bodies are positive for βCOP, SEC31, and ZW10 (Supplementary Figure S5A).

Q to A mutations in SNARE proteins have been reported to prevent cis-SNARE complex disassembly by N-ethylmaleimide sensitive fusion (NSF) after membrane fusion (Scales et al., 2001). Consistent with this, a super-resolution analysis also reveals that the large GFP–SNAP29^Q1Q2^ bodies are enriched in the SNARE disassembly factor NSF when compared with the occasional colocalization observed in GFP–SNAP29-expressing cells (Figures 5C,D). Because by preventing disassembly by NSF GFP–SNAP29^Q1Q2^ might stabilize four-helix bundles containing SNAP29, we next compared GFP–SNAP29 and GFP–SNAP29^Q1Q2^ immunoprecipitations using the GFP–Trap assay. Remarkably, we found that in GFP–SNAP29^Q1Q2^ immunoprecipitations the interaction with SEC22B is almost completely lost, while the binding with STX18 or STX5 is maintained (Figure 5E and Supplementary Figure S5B). Importantly, while GFP–SNAP29 immunoprecipitants also include the SNAREs USE1 and BNIP, which are known to associate with SEC22B and SXT18 for fusion of vesicles to the ER (Hirose et al., 2004; Nakajima et al., 2004), the levels of these are strongly reduced in GFP–SNAP29^Q1Q2^ immunoprecipitations (Figure 5E). These data indicate that SNAP29 might initially form complexes that only include STX18 or STX5. Importantly, SEC22B immunoprecipitates STX18 and GFP–SNAP29 in GFP–SNAP29-expressing cells, while less STX18 and no GFP–SNAP29^Q1Q2^ can be immunoprecipitated by SEC22B in GFP–SNAP29^Q1Q2^-expressing cells (Figure 5F). These data are consistent with the possibility that a complex might form between SNAP29 and STX18 and that SNAP29 is required to enhance the formation of a fusion complex containing SEC22B. Indeed when SNAP29 is depleted, SEC22B immunoprecipitates very low amounts of STX18 when compared with control cells (Figure 5G).

### Loss of SNAP29 in a Model of Human Neural Development

**FIGURE 6:**
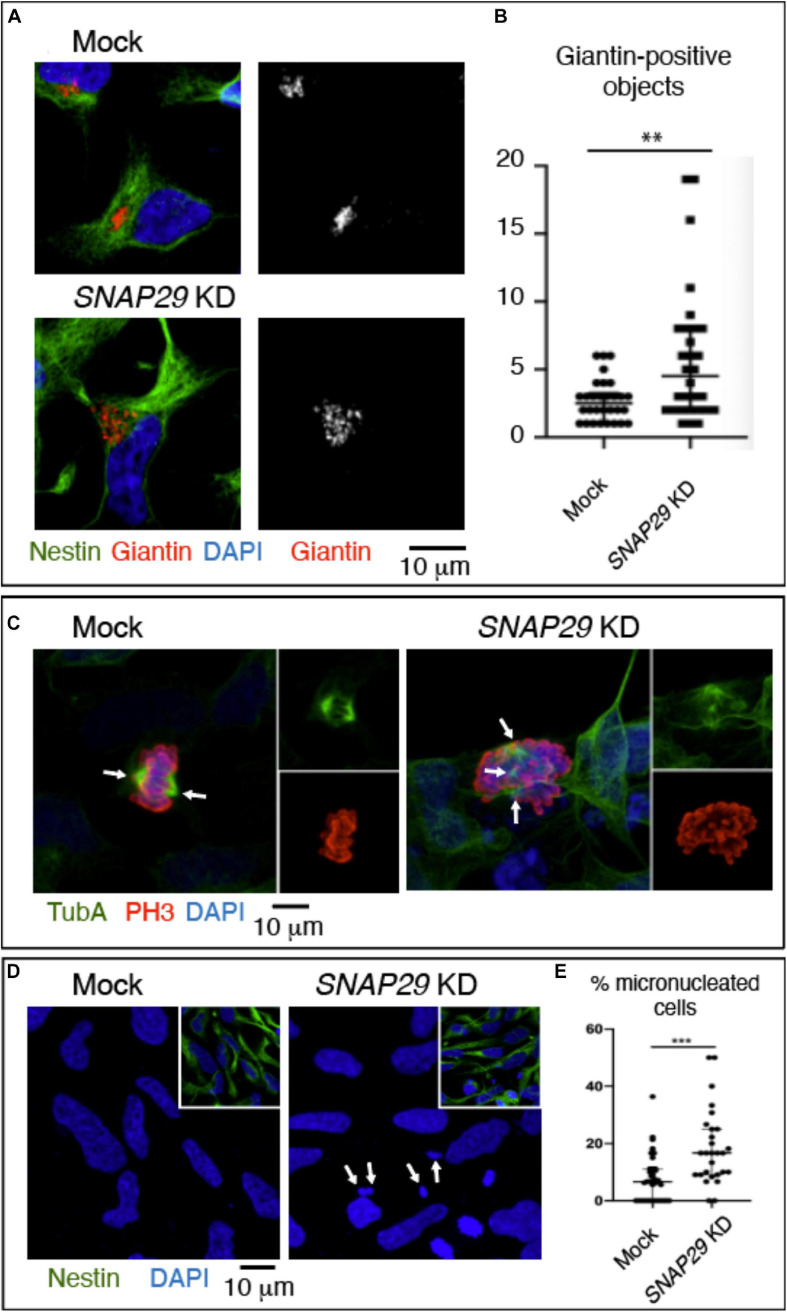
*SNAP29* depletion in neuroepithelial stem (NES) cells causes Golgi apparatus (GA) and spindle alteration and formation of micronuclei. **(A)** Maximal confocal projections of NES cells treated and stained as indicated. Depleted NES cells display GA fragmentation. **(B)** Quantification of the number of Giantin-positive objects. The median with interquartile range is shown, and the *p*-value is obtained by Mann–Whitney test. **(C)** Maximal confocal projections of NES cells treated and stained to detect α-tubulin and p-Histone3. The depleted NES cells in metaphase show an altered mitotic spindle. The arrows indicate spindle poles. **(D)** Maximal confocal projections of NES cells treated and stained as indicated. The depleted NES cells possess several micronuclei (arrows). **(E)** Quantification of the percentage of cells with at least one micronucleus. The median with interquartile range is shown, and the *p*-value is obtained by Mann–Whitney test.

To model the pathogenesis of CEDNIK in the developing neuro-epithelium, we took advantage of human NES cells. Upon depletion of *SNAP29* in NES, we observed alteration of the GA morphology (Figures 6A,B and Supplementary Figure S6A). In addition, *SNAP29*-depleted NES cells displayed spindle alterations (Figure 6C) and a mild impairment in mitotic progression (Supplementary Figure S6B). Furthermore, *SNAP29* KD NES cells often formed micronuclei, compared to mock-treated controls (Figures 6D,E). This evidence suggests that most cellular phenotypes associated with loss of SNAP29, including fragmentation of the GA, are likely to occur during neuro-epithelial development of CEDNIK patients.

## Discussion

**FIGURE 7:**
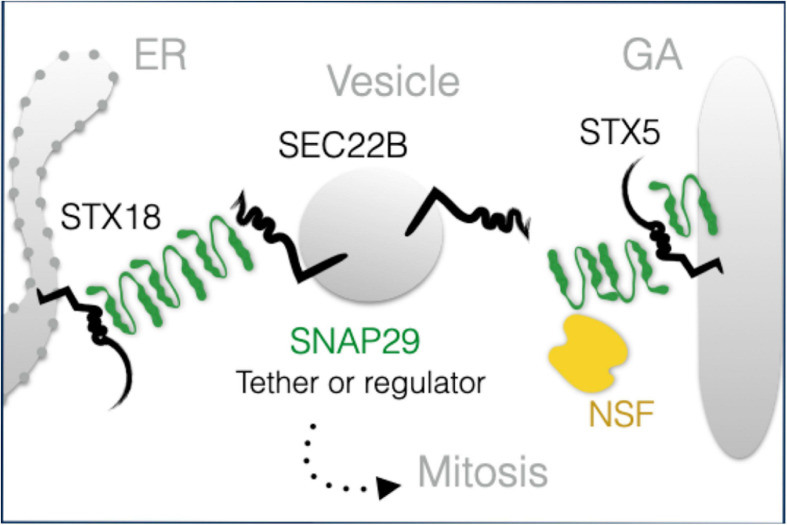
A model of SNAP29 activity at the endoplasmic reticulum (ER) and Golgi apparatus (GA). SNAP29 forms elongated structures that could assist the tethering of vesicles and/or that could regulate STX5/18 fusion competence. Some of SNAP29 are re-localized from the ER–GA area to form the outer kinetochore of mitotic chromosomes in prophase.

While the observed morphologic and functional GA alterations might be due to the indirect effects of SNAP29 depletion on endocytic, autophagic, or recycling trafficking, our localization and interaction data strongly suggest that SNAP29 acts directly with other SNAREs during GA trafficking. SNARE-mediated membrane fusion involves docking of R-SNAREs to receptor Q-SNAREs on target membranes with the formation of a highly structured four-helix bundle SNARE complex (Sutton et al., 1998). The paradigmatic model of SNARE complex formation is the one including combinations of a R-SNARE with a Qa-SNARE, a Qb-SNARE, and a Qc-SNARE [for a review, see Hong (2005)]. At the ER, such complex in HeLa cells is composed of the Qa-SNARE STX18, the R-SNARE SEC22B, and the Qb- and Qc-SNAREs USE1 and BNIP (Hatsuzawa et al., 2000; Hirose et al., 2004; Nakajima et al., 2004; Aoki et al., 2008). Our data indicate that an additional STX18 complex might include SNAP29. A similar complex containing STX5 in place of STX18 might be formed at the GA. Our super-resolution data, showing that exogenously expressed GFP–SNAP29 forms elongated and branched structures, suggest that these complexes might also include multimers of SNAP29. Based on Q to A changes in the 0-layer of GFP–SNAP29 resulting in the exclusion of SEC22B and the heavy recruitment of NSF, the ATPase that solubilizes cis-SNARE complexes (Weber et al., 2000), one possibility to be addressed in future studies is that elongated SNAP29 complexes might initially contact STX18 or STX5 and that their rearrangement, perhaps by NSF, might be required to engage COPI vesicles carrying SEC22B (Figure 7). Whether in such scenario SNAP29 acts as an unconventional tether or as a competitor of SEC22B for binding to STX18 or STX5 remains to be determined. SNAP29 might be uniquely suited to form elongated cytoplasmic structures because it is not stably associated with membranes and possesses a linker region between the SNARE domains that is distinct from that of paralogs SNAP25 and SNAP23. Thus, efforts should now focus on understanding whether such region allows a single SNAP29 molecule to be incorporated into two separate four-helix bundles, a prerequisite to form multimers. Consistent with this, impairment of NSF dissociation in yeast occurs only by Q to A mutations of Qa-SNAREs, but not of Qb-, Qc-, or R-SNAREs (Scales et al., 2001), suggesting that SNAP29 might behave in a four-helix bundle (also) as a Qa-SNARE.

Irrespective of the structure of Snap29-containing complexes, previous findings support the possibility that Snap29 might act to modulate the function of ER and GA SNARE complexes rather than solely engaging in fusion complexes. In fact, at the plasma membrane of neuronal cells, Snap29 has been reported to inhibit, rather than promote, membrane fusion (Su et al., 2001). Snap29 also does not rescue the loss of paralog Snap25 and possesses low propensity to form SNARE complexes or to be incorporated in stable SDS-resistant SNARE complexes (Steegmaier et al., 1998; Xu et al., 2014; Arora et al., 2017). Interestingly, Snap29 has been recently proposed to take part in a regulatory complex acting alongside the HOPS tethering complex in autophagosome–lysosome fusion (Matsui et al., 2018; Takáts et al., 2018). Future work is required to determine whether Snap29 acts in association with ER and GA tethers, for instance, the NRZ (Nag, Rint-1, Zw10) complex (Sönnichsen et al., 1998; Ren et al., 2009; Tripathi et al., 2009).

The impact of molecular perturbation of SNAP29 functions on human development are demonstrated by CEDNIK syndrome, characterized by a unique constellation of clinical manifestations including microcephaly, severe neurologic impairment, psychomotor retardation, failure to thrive, and facial dysmorphism as well as palmoplantar keratoderma and late-onset ichthyosis (Sprecher et al., 2005; Fuchs-Telem et al., 2011). Brain magnetic resonance imaging shows various degrees of cerebral dysgenesis, including absence of corpus callosum and cortical dysplasia. To test whether the cellular alterations are associated with the lack of SNAP29 relevant to early human neurodevelopment, we employed human NES cells, an *in vitro* model of long-term, self-renewing neuropotent stem cells (Onorati et al., 2016; Dell’Anno et al., 2018). Other forms of microcephaly have been successfully modeled in NES, such as that induced by mitotic impairment, centrosomal aberrations, and cell death upon ZIKV infection (Onorati et al., 2016). Thus, our data showing that depletion of *SNAP29* in NES cells produces GA fragmentation, spindle alterations, and impairment in mitotic progression with formation of micronuclei pave the way for generation of NES cells derived from induced pluripotent stem cells (iPSCs), which have been already used for mechanistic dissection of human genetic diseases of the CNS (Koch et al., 2011; Mertens et al., 2013). We envision that future analysis of phenotypes from CEDINK patient-derived NES might further elucidate the link between SNAP29 activity and neuroectodermal development.

## Materials and Methods

### Cell Cultures and Treatments

*Drosophila* Schneider-2 (S2) cells were cultured in Schneider medium (Thermo Fisher Scientific) supplemented with 10% fetal bovine serum (FBS) at 28°C. The mycoplasma-free HeLa cell line and the HeLa cell lines stably expressing ManII–SBP–GFP (Boncompain et al., 2012) were cultured in DMEM (Gibco) supplemented with 2 mM L-glutamine and 10% FBS at 37°C with 5% CO2. The HeLa cell line stably expressing E GFP–SNAP29 is a monoclonal line obtained after the transfection of pEGFPSNAP29 and clonal selection on 0.5 mg/ml G418. The stable GFP–SNAP29 HeLa cell line was cultured in DMEM (Gibco) supplemented with 2 mM L-glutamine, 10% FBS, and with the addition of 0.5 mg/ml G418 at 37°C with 5% CO2. For ManII–SBP–GFP trafficking from ER to GA, the cells were treated for 20 min in the presence of Biotin according to Boncompain et al. (2012).

The NES cells were derived from human iPSCs after a neural induction process *via* dual SMAD inhibition (Sousa et al., 2017). The NES cells were cultured, as previously described (Onorati et al., 2016; Dell’Anno et al., 2018), in NES medium including DMEM/F12 (Gibco #11330-032), with addition of B27 supplement (1:1,000, Invitrogen, #175040-44), N2 supplement (1:100, Gibco, #17502-048), 20 ng/ml FGF-2 (Gibco, #13256-029), 20 ng/ml EGF (Gibco, #PHG0311), 1.6 g/l glucose, 20 μg/ml insulin (Sigma, # I9278), and 5 ng/ml BDNF (R&D Systems Inc., #248-BD-01M). The cells were plated into dishes coated with poly-L-ornithine (0.01%, Sigma, #P4957), laminin (5 μg/ml, Invitrogen #23017-015), and fibronectin (1 μg/ml, Corning, #354008). Routinely, the NES cells were kept in proliferation until reaching confluency (0.5–1 × 10^5^ cells/cm^2^). The cells were expanded in NES medium and split 1:2–1:3 approximately every 5–7 days with 0.25% trypsin, adding 10 μM rock inhibitor (Y-27632, Stemgent, #04-0012) into the NES medium to increase cell viability. Half of the media was changed every 2 to 3 days to allow culture conditioning. All NES works were performed according to the NIH guidelines for the acquisition and distribution of human tissue for bio-medical research purposes and with approval by the human investigation committee and institutional ethics committee of each institute from which the samples were obtained. De-identified human specimens were provided by the Joint MRC/Wellcome Trust (grant #099175/Z/12/Z) Human Developmental Biology Resource^1^. Appropriate informed consent was obtained, and all available non-identifying information was recorded for each specimen. The tissue was handled in accordance with the ethical guidelines and regulations for the research use of human brain tissue set forth by the NIH^2^ and the WMA Declaration of Helsinki^3^.

### Fly Husbandry and Experiments

The flies were reared at 25°C in standard cornmeal food. The traffic-jam-Gal4 line to over-express in *Drosophila* follicle cells was provided by Veit Riechmann (University of Heidelberg). The UAS CFP-Snap29 was generated in Morelli et al. (2014). The list of genotypes for the experiment is in Supplementary Table 1.

### Immunostainings

The cells were fixed and stained as in Kobia et al. (2014). The following primary antibodies were used: chicken anti-GFP 1:1,000 (Abcam), mouse anti-Golgin97 1:100 (Invitrogen), rabbit anti-Giantin 1:1,000 (Bio Legend), rabbit anti-GM130 1:1,000 (cell signaling), rabbit anti-βCOP 1:1,000 (Invitrogen), mouse anti-SEC31 1:100 (BD Biosciences), rabbit anti-VAPB 1:1,000, rabbit anti-KDEL receptor (KDELR) 1:200 (a gift from A. DeMatteis), mouse anti-ERGIC53 1:1,000, rabbit anti-SEC22B 1:1,000 (SYSY), rabbit anti-STX5 1:1,000 (SYSY), mouse anti-STX18 (Santacruz); rabbit anti-NSF 1:1,000 (SYSY), DAPI 1:1,000 (Sigma); mouse anti-p-Histone3 1:2,000 (Abcam), rat anti-α-tubulin 1:100 (AbD Serotec), and mouse anti-Nestin 1:200 (R&D Systems Inc., #MAB1259). *Drosophila* wing discs, ovaries, and S2 cells were fixed and stained as in Morelli et al. (2016). The cells, disks, and ovaries were mounted on slides using Mowiol Mounting Medium. The following primary antibodies were used: chicken anti-GFP 1:1,000, rabbit (Abcam), anti-GM130 1:1,000 (Abcam), rabbit anti-Snap29 1:1,000 (Morelli et al., 2014), and mouse anti-Golgin84 1:20 (DSHB). Alexa-conjugated secondary antibodies (Invitrogen), rabbit Atto594 (Sigma), chicken Alexa488, rabbit Alexa 546, mouse Alexa647, and Phalloidin-TRITC (Sigma) were used. For all confocal imaging, we used a Leica microscope with × 40/NA 1.25 or × 63/NA 1.4 oil lenses and a Nikon A1 two-photon confocal microscope with × 40 or × 60 lenses. Super-resolution images were collected on a Leica TCS SP8 STED 3X microscope equipped with three depletion laser lines (592, 660, and 775 nm) and using a HCPL APO 100X/1.40 oil immersion objective. Images were acquired through the Software Leica LAS X and deconvolved with SVI Huygens Professional software. The images were edited with ImageJ and assembled with Adobe Illustrator.

### Electron Microscopy

Electron microscopic examination, EM tomography, and immune EM gold-labeling based on pre-embedding were performed as previously described (Beznoussenko and Mironov, 2015). In particular, for immune EM gold-labeling, cryosections were stained with the anti-β COP antibody 1:100 (Abcam ab2899) and anti-GFP (Abcam ab6556) for 2 h, washed six times with 0.1% bovine serum albumin in phosphate-buffered saline (PBS), and then incubated with 1:50 protein-A gold 5 and 10 nm (PAG10, CMC, Utrecht, The Netherlands) in blocking solution for 20 min at room temperature.

For CLEM, 00.5 × 105 growing GFPSNAP29-HeLa cells were plated on Matek previously coated with poly-D-Lysine (Sigma-Aldrich) and let adhere for 24 h. The cells were fixed with 4% PFA + 0.05% glutaraldehyde in Hepes (0.15 M) adjusted to pH 7.2–7.4 for 5 min and then fixed again with 4% PFA in Hepes (0.15 M) adjusted to pH 7.2–7.4 three times for 10 min. The cells were quickly washed three times with Hepes (0.2 M) and imaged. Imaging was performed on a Leica TCS SP5 laser confocal scanner mounted on a Leica DMI 6000B inverted microscope equipped with a HC PL FLUOTAR × 20/0.5NA and a HCX PL APO × 63/1.4 NA oil-immersion objective and driven by Leica LAS AF software. The images were edited with ImageJ.

### Protein Extraction, Western Blots, and Immunoprecipitations

The cells were collected, homogenized, and incubated for 20 min on ice in 1 mM Tris–HCl, 150 mM NaCl, 5 mM EDTA, 1% Triton X-100, 1% deoxycholate, 0.1% SDS, and protease inhibitors 1:200 (Cal-biochem). The lysates were cleared by centrifugation. The supernatants were recovered and quantified, separated by SDS–PAGE, and transferred to nitrocellulose by standard methods. The primary antibodies used were rabbit anti-SNAP29 1:500 (Morelli et al., 2016), chicken anti-GFP 1:1,000 (Abcam), mouse anti-Vinculin (1:10,000), mouse anti-STX18 1:500 (Santa Cruz), and mouse anti-αtubulin 1:8,000 (Cell Signaling #3873), rabbit anti-STX5 1:1,000, rabbit anti-SEC22B 1:1,000, rabbit anti-USE1 1:500, rabbit anti-BNIP 1:500, and rabbit anti-STX18 1:500 (all from SYSY), rabbit anti-Snap29 1:1,000 (Morelli et al., 2014), and mouse anti-HA 1:500 (Covance). The secondary antibodies used were anti-rabbit and anti-mouse 10,000 (Amersham), anti-chicken 1:1,000 (Invitrogen), and anti-mouse Trueblot 1:1,000 (Roche). Immunoblots were visualized with SuperSignal West Pico/Femto Chemiluminescent Substrate (Bio-Rad) using Chemidoc (Bio-Rad). HeLa and S2 cell immunoprecipitations were performed in high salt JS buffer (Tris–HCl pH 7.6, NaCl 150 mM, glycerol 20%, 0.5% NP-40, MgCl2 2 mM, Na pyrophosphate 0.1 M pH 7.5, PMSF 0.1 M in ethanol, Na vanadate 0.5 M pH 7.5 in Hepes, NaF 0.5 M) with addition of protease inhibitors 1:200 (Calbiochem). The antibodies used were rabbit anti-SNAP29 (Morelli et al., 2016), mouse anti-STX18 1:500, rabbit anti-STX5 1:1,000, rabbit anti-SEC22b 1:1,000 all from SYSY, rabbit anti-Snap29 (Morelli et al., 2014), and mouse anti-HA (Covance). Then, 2 ug of antibodies was used for 200 ug of protein extract. Immunoprecipitation was performed using Sepharose ProteinG (Invitrogen), and precipitation of GFP tagged protein was performed using the GFPTrap system (Chromotek).

### siRNA Silencing

For *SNAP29*, *STX5*, *STX18*, and *SEC22B* knockdown, we performed a reverse transfection with Lipofectamine RNAi Max (Thermo Fisher) according to the manufacturer’s instruction. We used *SNAP29* (D-011935-04-0005) and *STX18* (E-020624-00-0005) siRNA (Dharmacon). To evaluate ManII–SBP–GFP trafficking, we used *SEC22B* (EMU019661), *STX18* (EHU025321), and *STX5* (EHU012041) EasyRNA (Sigma). Cells were collected at different time points (mostly 48 and 72 h) after transfection and processed for further analysis. The control transfections are mock transfections performed with the same procedure as detailed above in the absence of siRNA.

For reverse transfection of NES cells, RNAi duplex-Lipofectamine^TM^ RNAiMAX (Invitrogen) was prepared as follows: for each six-well plate sample, 150 μl of Opti-MEM Medium, 6 μl of RNAiMAX, and 9 μl of 10 μM siRNA for SNAP29 were directly added into the wells, while only the optimum and RNAiMAX reagent were added in the control wells. The plate was incubated for 10 min at room temperature. Meanwhile, cells were trypsinized, and 500,000 cells were diluted in 2 ml of NES medium without antibiotics. After the incubation, 2 ml of cell suspension was added in each well. The cells were incubated for 72 and 96 h at 37°C in a CO_2_ incubator before analysis. For Western blotting, wells were washed with PBS, 80 μl of RIPA buffer + inhibitor was added directly in the well, and the cells were scraped. The plate was kept rocking at 4°C for 30 min. Then, the cells were spun down, and the supernatant was used to perform the Bradford protein assay and Western blot.

### Transfection of GFP-Tagged SNAP29 Forms

The human SNAP29 cDNA encoding a siRNA-resistant RNA and the mutant SNAP29^Q1Q2^ forms were generated as described in Morelli et al. (2016). SNAP29 and SNAP29^Q1Q2^ were then inserted into pEGFP-C1 within *Eco*RI/*Bam*HI restriction enzyme sites. For rescue or over-expression experiments, a mix composed of the relevant vector alone or mixed with the siRNA specific for SNAP29 and Lipofectamine 2000 was prepared following the manufacturer’s instruction (Invitrogen). Cells were collected at 6 or 24 h after transfection.

### Measurements and Statistics

Quantification of the GA major/minor axis was performed using the ImageJ plugin Fit Ellipse, which splits binary objects which could be approximated by an ellipse, giving the measurement of a major and a minor axis, respectively. Quantification of cisternal width has been performed using ImageJ by drawing a line across each cisterna in the central part of the GA and by recording the length of the line relative to the scale bar. Quantification of Golgi objects and Golgi area was performed with ImageJ by drawing a mask around the Golgi signal (Golgin97 or Giantin) and counting the number of identified objects. Quantification of MannII-SBP-EGFP was performed with ImageJ by drawing a region of interest (ROI) around the Giantin signal to identify the Golgi units. A second ROI identified the whole cell using the cortical phalloidin signal (not shown in the figure). The fluorescence intensity of the MannII-SBP-EGFP signal within the Golgi area was measured using the first ROI (labeled Giantin-positive GFP in the quantification), while the total fluorescence intensity of the MannII-SBP-EGFP was measured using the second ROI (labeled total GFP in the quantification). All experiments have been repeated at least three times, and for each experiment, at least 20 cells from each sample have been analyzed. Statistical analysis of each quantification (indicated in the figure legends) was performed with Prism.

## Data Availability Statement

The original contributions presented in the study are included in the article/Supplementary Material, further inquiries can be directed to the corresponding author/s.

## Author Contributions

EM designed, performed, quantified experiments, and wrote the initial draft of the study. ES performed the Drosophila experiment. EP and MO performed the NES experiments. GB and AM conducted the EM analyses. FC provided prepared reagents and material for experiments in cells and in Drosophila. MG contributed to the super-resolution microscopy. TV coordinated the team, supervised the study and wrote the manuscripts with inputs from all authors.

## Conflict of Interest

The authors declare that the research was conducted in the absence of any commercial or financial relationships that could be construed as a potential conflict of interest.

## Footnotes

1 www.hdbr.org

2 http://bioethics.od.nih.gov/humantissue.html

3 http://www.wma.net/en/30publications/10policies/b3/index.html

## Supplementary Material

Supplementary Figure 1 **(A–D)** Single confocal sections of mock and SNAP29 KD HeLa cells or, in addition, over-expressing the indicated transgenes, stained as indicated. **(E)** Quantification of the number of Golgin97-positive objects. The mean with standard error of the mean is shown, and the *p*-value is obtained by one-way ANOVA with Tukey’s multiple-comparisons analysis. The Golgi apparatus (GA) alterations upon SNAP29 depletion are rescued expression of GFP–SNAP29. **(F)** Quantification of the width of GA cisternae of EM sections such as those shown in Figures 1H–J. The median with interquartile range is shown, and the *p*-value is obtained by Mann–Whitney test.

Supplementary Figure 2 **(A,B)** Single section of a portion of *Drosophila* egg chamber stained to reveal endogenous Snap29 **(A)** or over-expressing CFP-Snap29 **(B)**, stained as indicated. **(C)** Immunoblotting of total proteins from CCD-1109 fibroblast protein extracts with the indicated antibody and related input. **(D)** Maximal confocal projections of CCD-1109 fibroblast treated and stained as indicated. Depleted cells show Golgi apparatus alteration.

Supplementary Figure 3 **(A,B)** Single confocal sections of HeLa cells stably expressing ManII–SBP–GFP, treated and stained as indicated. The EGFP pattern has been imaged before the addition of biotin (no biotin), 20 min after addition of biotin (20 min biotin), or 1 h after the addition of biotin (60’ biotin). SNAP29 depletion delays trafficking from the endoplasmic reticulum to the Golgi apparatus. **(C)** Immunoblotting of total protein extracts with antibodies recognizing the indicated proteins. HeLa cells were depleted as indicated. The asterisk indicates an unspecific band recognized by the anti-SNAP29 antibody. **(D)** Quantification of the number of Giantin-positive objects in the indicated sample. The mean with standard error of the mean is shown, and the *p*-value is obtained by one-way ANOVA with Tukey’s multiple-comparisons analysis.

Supplementary Figure 4 **(A)** Single sections of HeLa cells over-expressing GFP–SNAP29 for 6 h stained as indicated and acquired by stimulated emission depletion microscopy. The dashed and the continuous lines delimit the nucleus and the plasma membrane, respectively. The yellow arrows indicate points of co-localization between GFPSNAP29 and endoplasmic reticulum and Golgi apparatus SNAREs.

Supplementary Figure 5 **(A)** Single sections of HeLa cells over-expressing GFP–SNAP29 for 6 h stained as indicated and acquired by stimulated emission depletion microscopy. The dashed and the continuous lines delimit the nucleus and the plasma membrane, respectively. **(B)** Immunoblotting with the indicated antibodies of proteins immunoprecipitated using GFP Trap from protein extracts of HeLa cells expressing the indicated transgenes and related inputs and supernatants.

Supplementary Figure 6 **(A)** Immunoblotting of total proteins from neuroepithelial stem (NES) cell protein extracts to detect α-tubulin and p-Histone3. **(B)** Maximal confocal projections of NES cells treated and stained as indicated. *SNAP29*-depleted NES cells display a slightly increased mitotic index at 72 h.
