# Supplementary figures and images for: Activity of the SNARE Protein SNAP29 at the Endoplasmic Reticulum and Golgi Apparatus
Source: Front Cell Dev Biol. 2021 Feb 18;9:637565. doi: 10.3389/fcell.2021.637565 (PMC7945952; doi:10.3389/fcell.2021.637565)

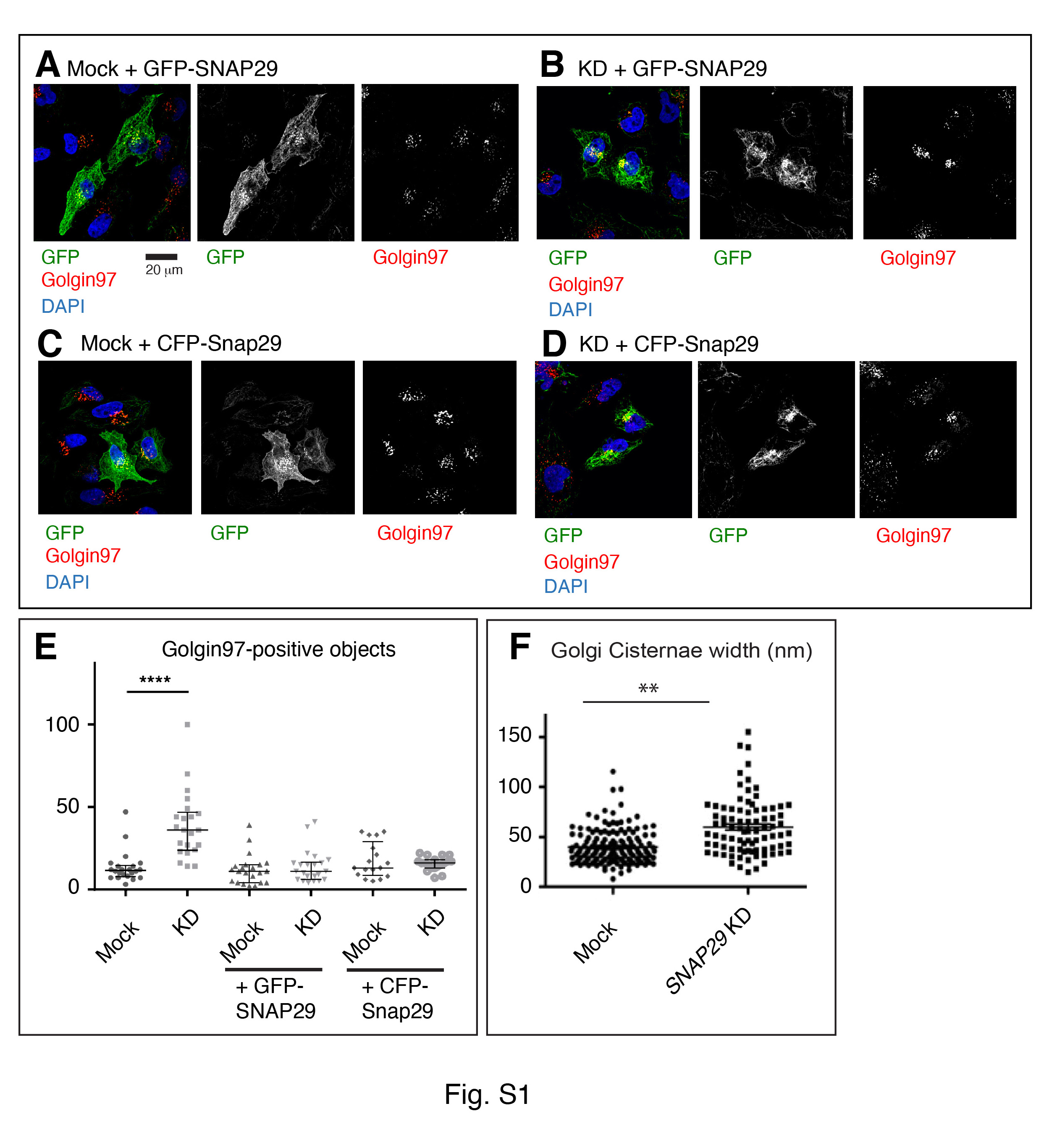

Supplement: Supplementary Figure 1 — (A–D) Single confocal sections of mock and SNAP29 KD HeLa cells or, in addition, over-expressing the indicated transgenes, stained as indicated. (E) Quantification of the number of Golgin97-positive objects. The mean with standard error of the mean is shown, and the p-value is obtained by one-way ANOVA with Tukey’s multiple-comparisons analysis. The Golgi apparatus (GA) alterations upon SNAP29 depletion are rescued expression of GFP–SNAP29. (F) Quantification of the width of GA cisternae of EM sections such as those shown in Figures 1H–J. The median with interquartile range is shown, and the p-value is obtained by Mann–Whitney test. [file Image_1.jpg]

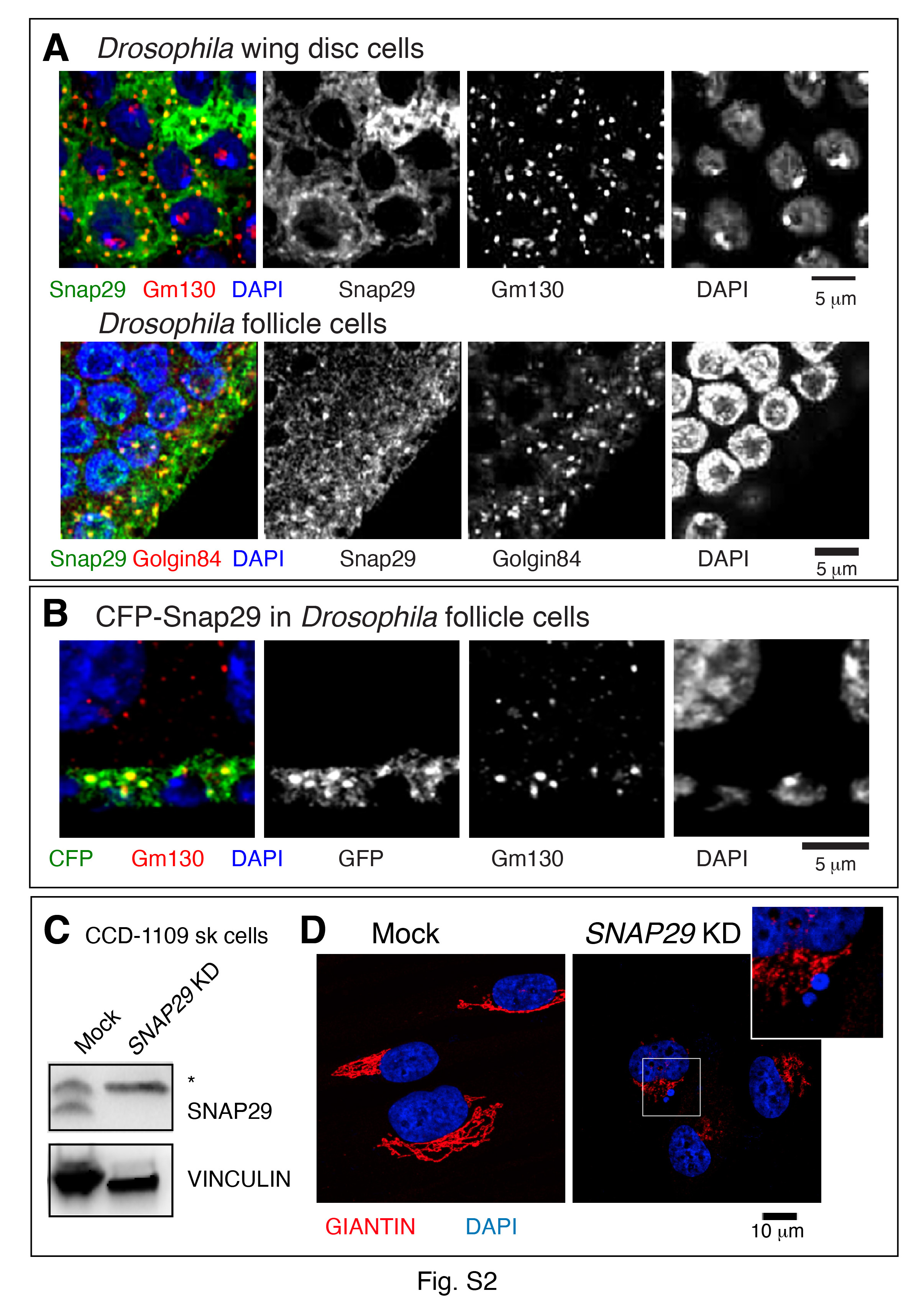

Supplement: Supplementary Figure 2 — (A,B) Single section of a portion of Drosophila egg chamber stained to reveal endogenous Snap29 (A) or over-expressing CFP-Snap29 (B), stained as indicated. (C) Immunoblotting of total proteins from CCD-1109 fibroblast protein extracts with the indicated antibody and related input. (D) Maximal confocal projections of CCD-1109 fibroblast treated and stained as indicated. Depleted cells show Golgi apparatus alteration. [file Image_2.jpg]

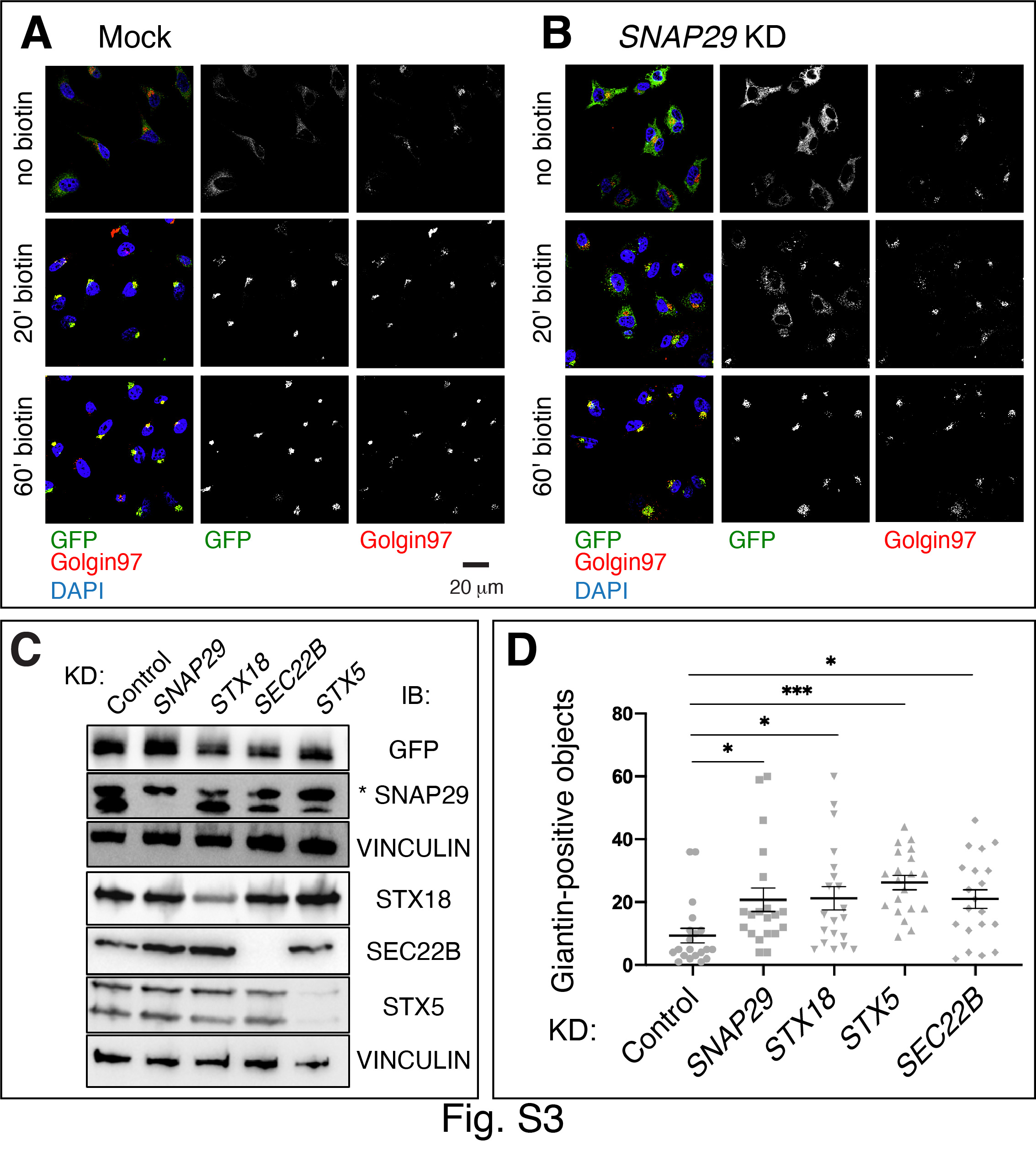

Supplement: Supplementary Figure 3 — (A,B) Single confocal sections of HeLa cells stably expressing ManII–SBP–GFP, treated and stained as indicated. The EGFP pattern has been imaged before the addition of biotin (no biotin), 20 min after addition of biotin (20 min biotin), or 1 h after the addition of biotin (60’ biotin). SNAP29 depletion delays trafficking from the endoplasmic reticulum to the Golgi apparatus. (C) Immunoblotting of total protein extracts with antibodies recognizing the indicated proteins. HeLa cells were depleted as indicated. The asterisk indicates an unspecific band recognized by the anti-SNAP29 antibody. (D) Quantification of the number of Giantin-positive objects in the indicated sample. The mean with standard error of the mean is shown, and the p-value is obtained by one-way ANOVA with Tukey’s multiple-comparisons analysis. [file Image_3.jpg]

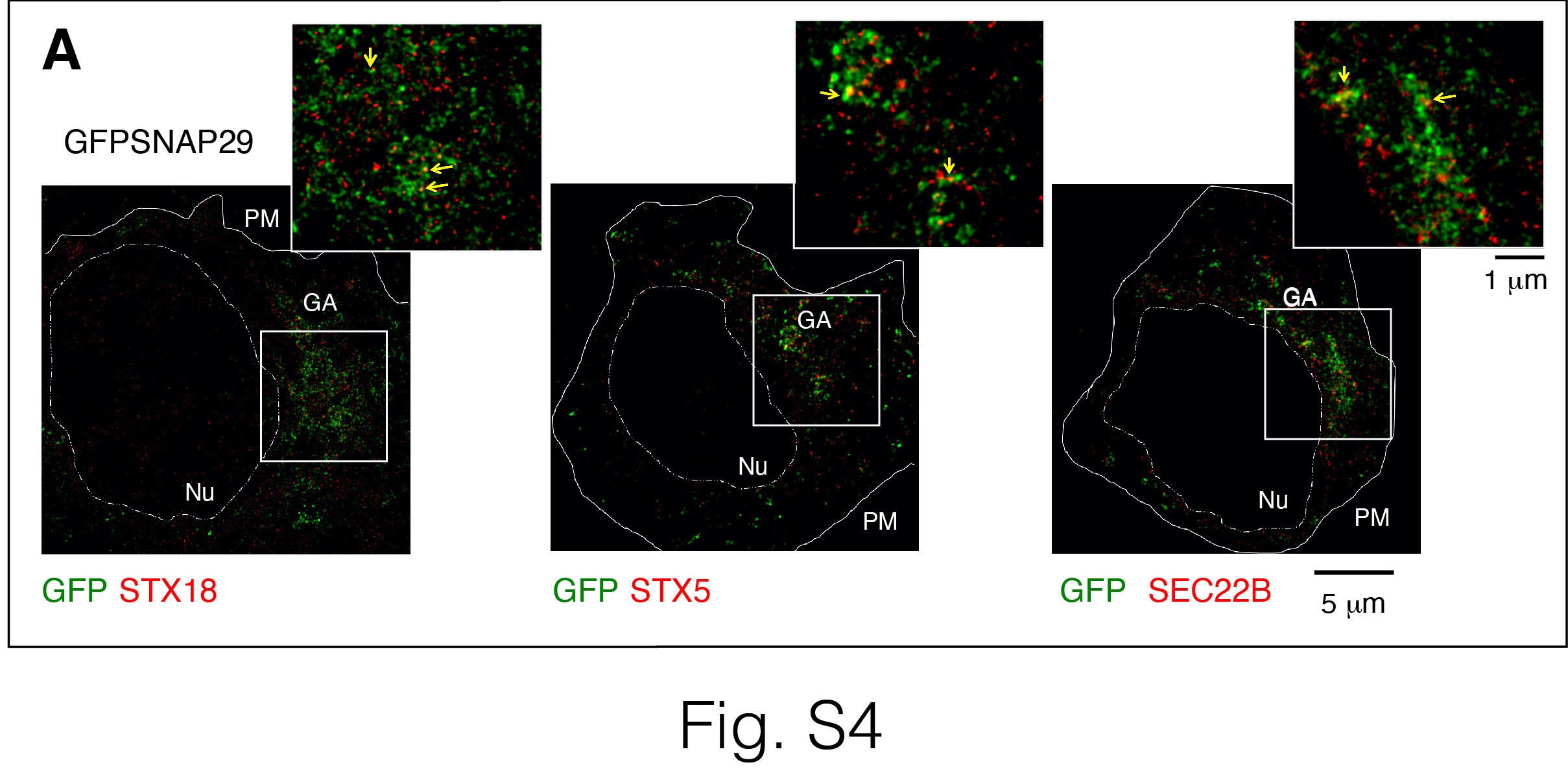

Supplement: Supplementary Figure 4 — (A) Single sections of HeLa cells over-expressing GFP–SNAP29 for 6 h stained as indicated and acquired by stimulated emission depletion microscopy. The dashed and the continuous lines delimit the nucleus and the plasma membrane, respectively. The yellow arrows indicate points of co-localization between GFPSNAP29 and endoplasmic reticulum and Golgi apparatus SNAREs. [file Image_4.jpg]

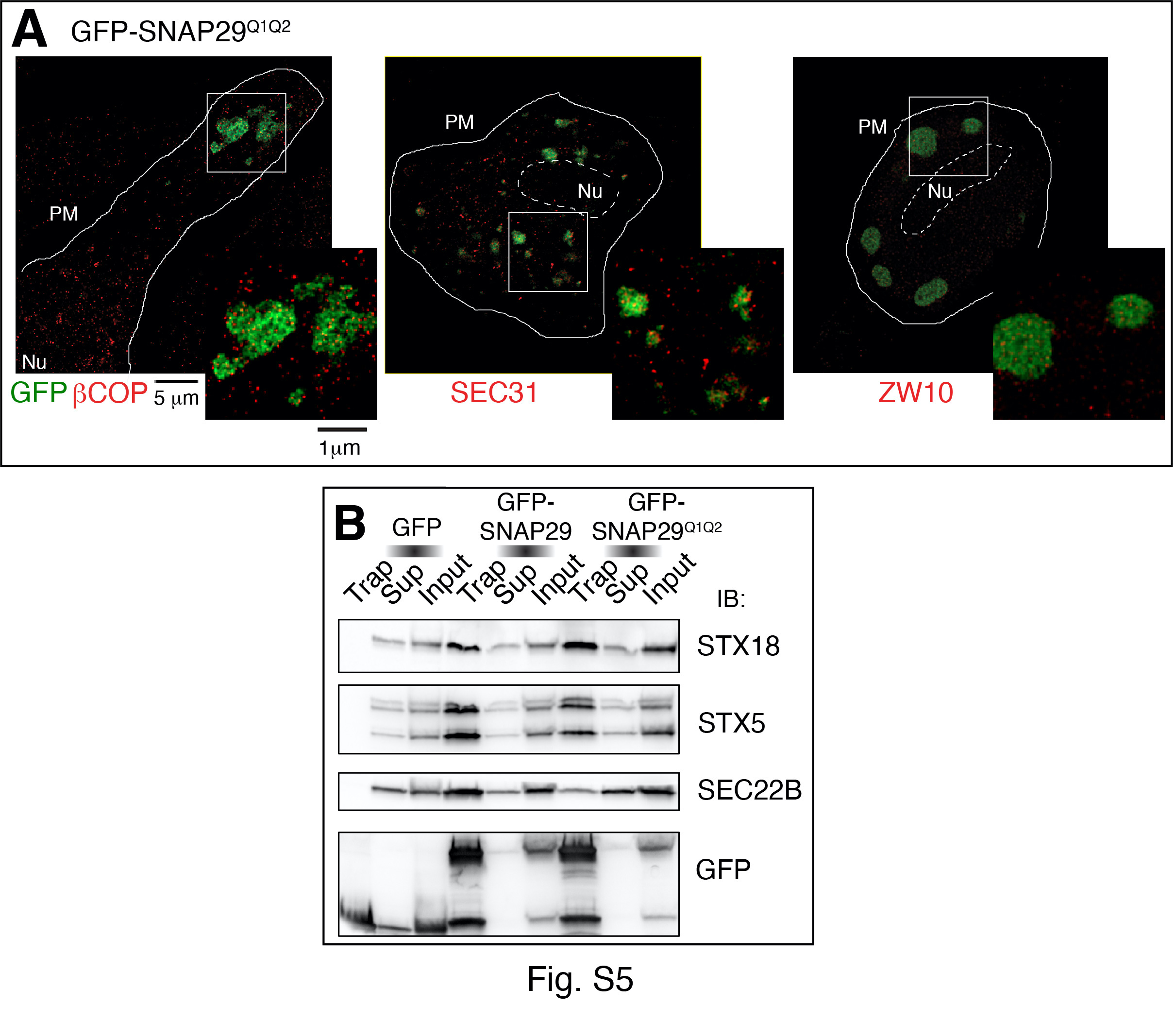

Supplement: Supplementary Figure 5 — (A) Single sections of HeLa cells over-expressing GFP–SNAP29 for 6 h stained as indicated and acquired by stimulated emission depletion microscopy. The dashed and the continuous lines delimit the nucleus and the plasma membrane, respectively. (B) Immunoblotting with the indicated antibodies of proteins immunoprecipitated using GFP Trap from protein extracts of HeLa cells expressing the indicated transgenes and related inputs and supernatants. [file Image_5.jpg]

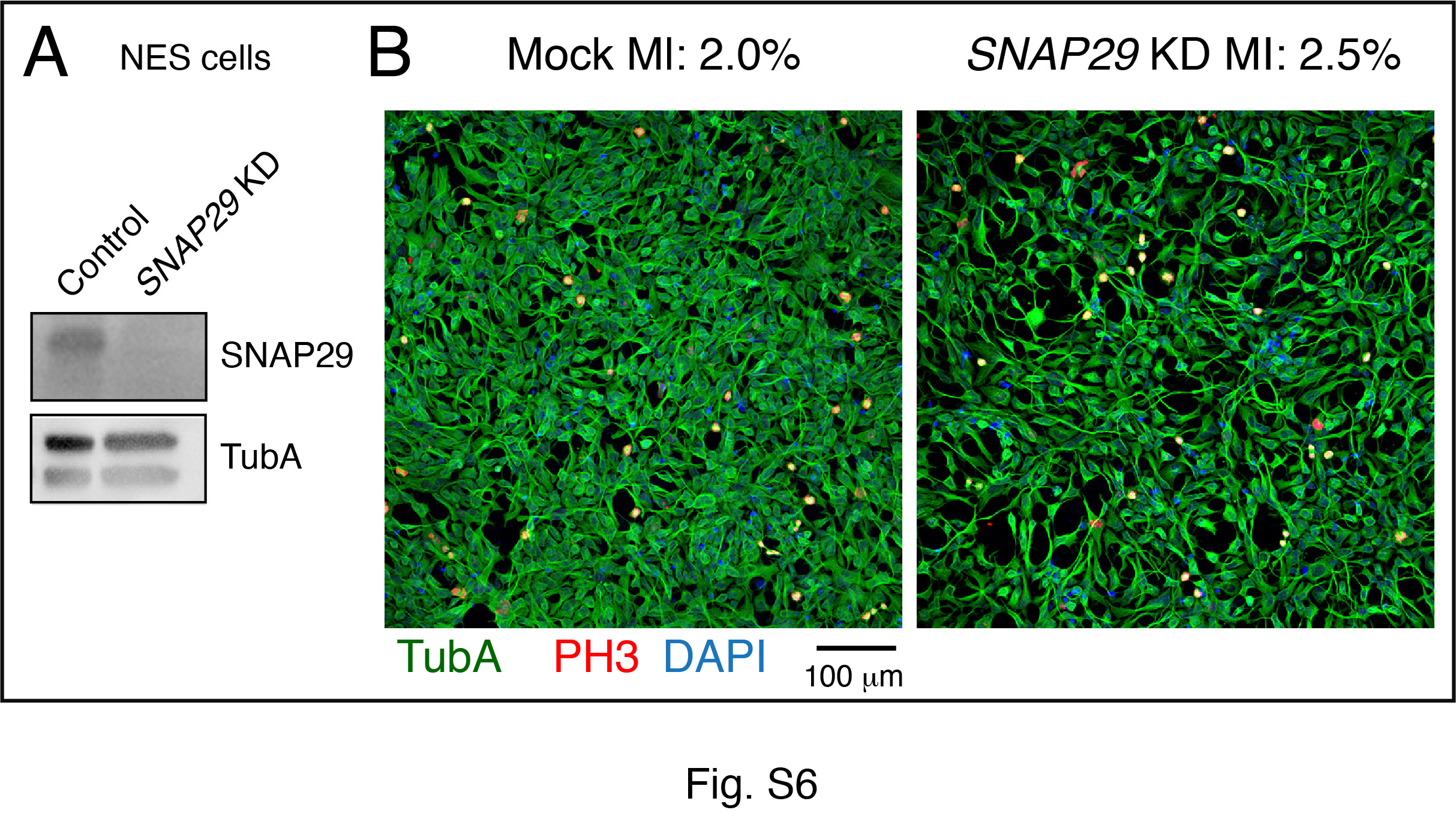

Supplement: Supplementary Figure 6 — (A) Immunoblotting of total proteins from neuroepithelial stem (NES) cell protein extracts to detect α-tubulin and p-Histone3. (B) Maximal confocal projections of NES cells treated and stained as indicated. SNAP29-depleted NES cells display a slightly increased mitotic index at 72 h. [file Image_6.jpg]
